# Unusual Manifestation of Benign Squamous Papilloma of the Uvula: A Case Report and Review of Literature

**DOI:** 10.7759/cureus.6716

**Published:** 2020-01-21

**Authors:** Khalid Aldhafeeri, Mohammed Alshaikh, Fawziyah Kilany, Saud AlKhaldi, Abdullah Alamri

**Affiliations:** 1 Otorhinolaryngology Head and Neck Surgery, Royal Commission Hospital, Jubail, SAU; 2 Otolaryngology, ENT and Cochlear Implant Center, Royal Commission Hospital, Jubail, SAU; 3 Otolaryngology, College of Medicine, Royal Commission Hospital, Jubail, SAU; 4 Otolaryngology Head and Neck Surgery, King Fahad General Hospital, Jeddah, SAU

**Keywords:** squamous papilloma, hpv, oral cavity, pharynx, larynx, epiglottis

## Abstract

Squamous papilloma is an exophytic overgrowth of the soft tissue that is associated with human papillomavirus infection. It is rarely reported in the literature and uncommonly located on the uvula. We report a rare case of a squamous papilloma located in the uvula. Despite the small size of the tumor, the patient complaints were significant to mass-related symptoms. In addition, related literature was reviewed and results were discussed.

## Introduction

Squamous papilloma is an exophytic overgrowth and projection of the soft tissue associated with human papillomavirus (HPV), with the function of the surrounding structures spared. It is usually benign and asymptomatic, appears as pedunculated, sessile or verrucous, and usually depends on its location [1,2]. Squamous papilloma occurs most commonly on the tonsils and on the base of the tongue, and to a lesser extent on the hard palate, tip of the tongue, gums, epiglottis, pharynx, and uvula. It accounts for the minority of papilloma presentations. While squamous papilloma is usually asymptomatic, when there is substantial overgrowth it could present with some discomforting symptoms such as dysphagia, globus sensation, dry cough, and throat clearing. Infrequently, more serious life-threatening complications of advanced manifestation may occur, such as airway obstruction [3,4]. Squamous papilloma is usually diagnosed during adulthood, predominantly in the females [5]. A provisional diagnosis is made within a clinical setting, but definitive diagnosis requires histopathology. Squamous papillomas usually have an absolute histopathological feature such as fibrovascular cores surrounded by stratified squamous epithelium, finger-like projections, and koilocytes [6].

## Case presentation

**Figure 1:**
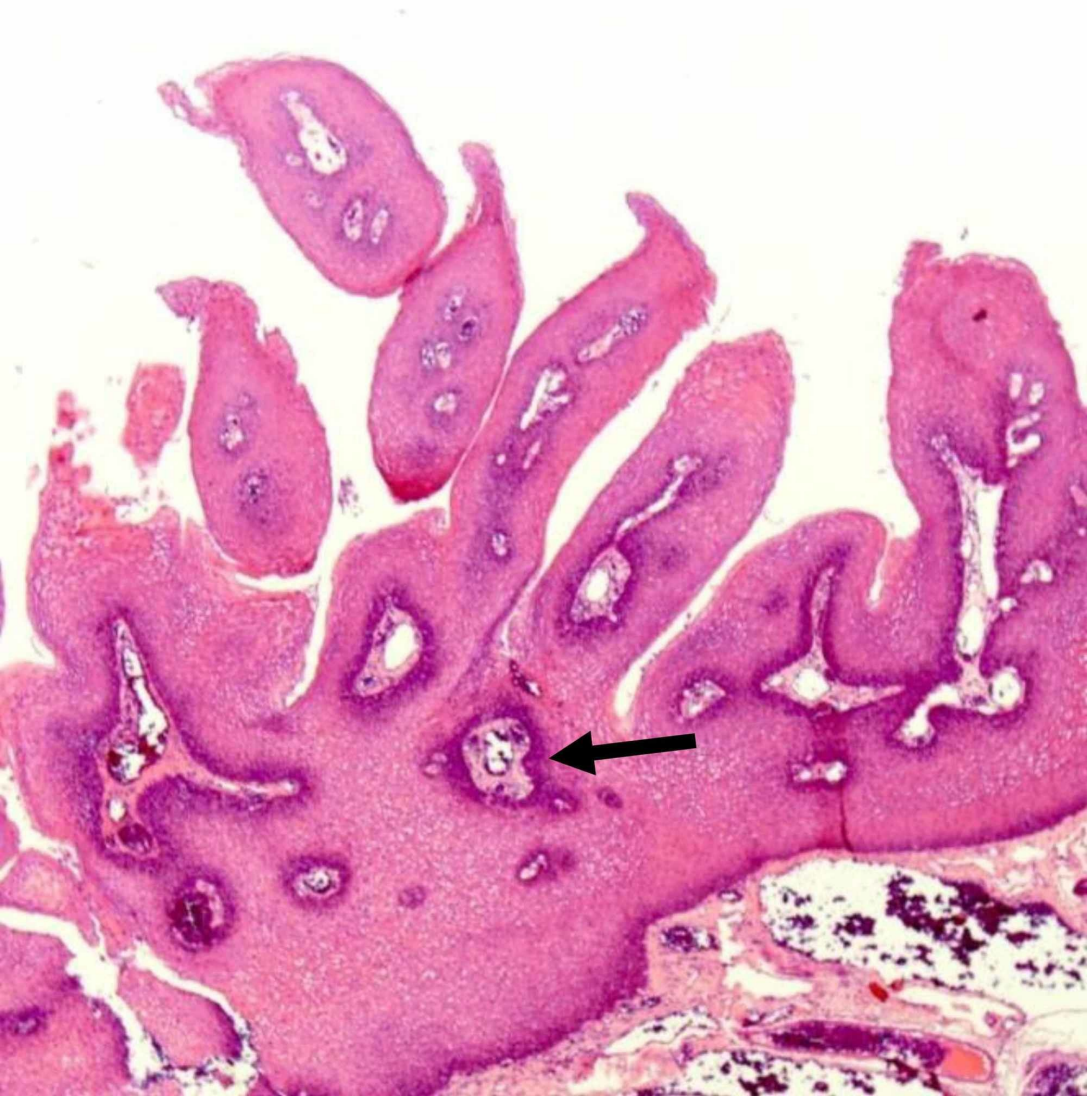
Low-power image shows exophytic lesion with finger-like papillary projections, containing thin fibrovascular cores, lined by benign stratified squamous epithelium.

**Figure 2:**
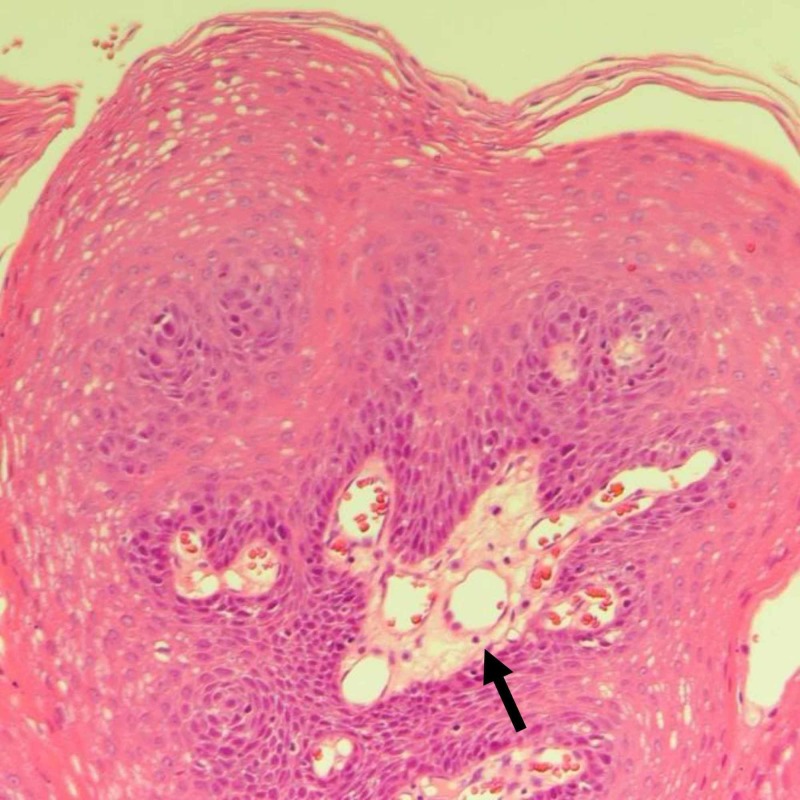
High-power image shows supporting fibrovascular cores, focal surface parakeratosis, lined by benign stratified squamous epithelium.

An 18-year-old female, otherwise healthy, presented with a five-month history of dysphagia. She also reported a choking sensation, globus sensation, and frequent throat clearing. There was no history of fever, throat pain, or infectious symptoms. Constitutional symptoms were negative. Upon throat examination, a fine strand of tissue over 0.5 cm in length was found to extend from uvular tip inferiorly, ending in a small disc-like pedunculated mass (0.5 x 0.5 x 0.5 cm); the base of the uvula appeared to be clean. Excision was simple with the use of electrocautery, and the specimen was sent to the histopathology laboratory. Histopathologic evaluation showed papillary projections lined by hyperplastic squamous epithelium around fibrovascular cores (Figure 1). The hyperplastic squamous epithelium is composed of elongated hyperchromatic nuclei with eosinophilic cytoplasm (Figure 2) suggestive of benign squamous papilloma. Four weeks after the surgical excision, normal healing and normal restoration of the uvular shape were seen.

## Discussion

An extensive review of the literature was conducted utilizing the Boolean operator of the PubMed database search engine. The following MeSH terms were used: squamous, papilloma, HPV, uvula, oral cavity, pharynx, larynx, epiglottis. A total of eight related studies were found and analyzed (Table 1). The general analysis of the literature review revealed the following: mean age of patients was 33.33 years, with a male-to-female ratio of 5:4. In terms of location, three were on the uvula, three on the hard palate, one on the buccal mucosa, one on the tongue, one on the vulva, and one on the epiglottis. Six were pedunculated, one was sessile, one was verrucous, and one was not specified.

**Table 1:** Review of related literature.

Reference number	Age	Gender	Presentation	Site	Size	Histopathological findings	Gross appearance
[1]	22	Female	Dysphagia, globus sensation, frequent throat clearing, dry cough, excess mucous, and heartburn	Uvula	1.5 cm	Multiple squamous lined papillary frond containing fibrovascular cores	Pedunculated
[2]	10	Male	Dysphagia, and a choking sensation while eating solid food	Uvula	3 x 2 cm	Finger-like projections and central vascular zone surrounded by stratified squamous epithelium	Pedunculated
[3]	43	Male	Incidental finding (airway obstruction)	Uvula	0.8 x 0.9 x 0.5 cm	None	Pedunculated
[4]	14	Male	Painless overgrowth	Hard palate	3 x 3 cm	Finger-like projection and central vascularization	Sessile
[5]	10	Female	Discomforting, occasionally painful overgrowth, that interferes with biting	Hard palate	1.5 cm	Proliferation in the form of finger-like projections containing a thin connective tissue core and koilocytes	Pedunculated
[6]	Case 1: 65	Male	Case 1: assessment of a mass	Case 1: buccal mucosa	Case 1: 2.5 x 1 cm	Case 1: pushing border, and minimal cytological atypia	Case 1: Verrucous
	Case 2: 44	Male	Case 2: painless mass	Case 2: hard palate	Case 2: 5 x 3 mm	Case 2: papillary mucosal mass surrounded by parakeratinized stratified squamous epithelium of varying thickness, with a papillary surface	Case 2: pedunculated
[7]	65	Female	Dyspepsia and heartburn	Suprahyoid epiglottis	1 cm	Central fibrovascular cores covered by a benign keratinizing squamous epithelium	Pedunculated
[8]	27	Female	Two painless masses	One on the anterior part of the tongue, and the other on the vulva	On the tongue: 1 cm On the vulva: 2 cm	Epithelium irregularly thickened by acanthosis and papillomatosis with an area of parakeratosis, and identification of koilocytes	Not specified

According to the World Health Organization’s current classification, oral squamous papilloma is a benign exophytic, hyperplastic, localized proliferation with a verrucous or cauliflower-like morphology. Its base may be pedunculated or sessile [7].

The squamous papilloma usually presents as asymptomatic for years, but if it is symptomatic, it could present with some or all of the following related symptoms according to its location and size: dysphagia, sore throat, odynophagia, voice quality change, referred otalgia, neck mass, globus sensation, dysarthria, trismus, a palpable discomforting mass, and decreased tongue mobility [3].

Whether the mass is symptomatic or asymptomatic mainly depends on its nature, size, shape, consistency, stage, site, and risk factors of the patient. In this case, the patient’s mass was considered small (i.e., 0.5 x 0.5 x 0.5 cm), though her symptoms were severe enough to cause her discomfort. This does not follow the usual presentation; however, other causes of the symptoms were ruled out.

The true prevalence of squamous papilloma in the oral cavity and oropharynx is not well known; nevertheless, it must not be neglected: recent report describes an incidence of 6.39% of squamous papilloma among all oral benign tumors, predominantly in females, the majority in their second, third, and fourth decades. Local studies to define the magnitude of squamous papilloma in Saudi Arabia are notably lacking [5].

Squamous papilloma can be found anywhere in the upper aerodigestive tract, most commonly on the tonsils and on the base of the tongue. They are found to a lesser extent on the hard palate, tip of the tongue, gums, epiglottis, pharynx, and uvula [3].

Such lesions are mostly associated with HPV infection, most commonly P16, which is a dsDNA virus that infects stratified squamous epithelium. Moreover, it affects both oncogenes E6 and E7, which are responsible for malignancies in both the anogenital and head and neck areas. E6 binds and inactivates p53 TSG, while E7 binds and inactivates the retinoblastoma protein, leading to the release of the E2F transcription factor, thus causing cell cycle progression [3].

Histologically finger-like projections of fibrovascular tissue covered by hyperkeratotic benign stratified squamous epithelium and a marked granular cell layer are observed. Koilocytes are usually seen in lesions of short time of growth. In addition, small lymphocytic inflammation foci can appear at the base of the lesion, which are often not seen unless the lesion is subjected to repeated trauma or other irritations [6].

Provisional diagnosis is made within the clinical setting using various techniques such as immunohistochemistry and molecular analyses. However, confirmatory diagnosis requires a biopsy and further histopathological evaluation [8]. Differential diagnoses could include verrucous carcinoma, squamous papilloma, lymphoma, or lymphoepithelioma [3].

The treatment of choice is complete surgical excision including the base of the lesion and a small marginal area to prevent recurrence. The use of laser treatments has also been proposed to eliminate oral squamous cell papilloma, rather than the surgical scalpel [9].

Bivalent and quadrivalent HPV vaccines have been included in the national immunization programs of at least 26 nations. The recent progress in biotechnology, immunotherapy, molecular biology, and recombinant DNA technology, along with alternative and complementary medicinal systems, has created novel methods and valuable opportunities to design and develop effective prophylactic and therapeutic vaccines and medication to effectively counter HPV [10].

Minimal progression and reduced local-regional failure indicate a better prognosis. There are many types of HPV, but in the case of squamous papilloma, HPV type 16 is the primary causative agent [3].

## Conclusions

Of the nine cases of squamous papilloma found based on extensive literature review, squamous papilloma on the uvula is uncommon and rarely reported. In addition, despite the small size of the tumor, the patient complaints were significant to mass-related symptoms.
